# Untapped aspects of mass media campaigns for changing health behaviour towards non-communicable diseases in Bangladesh

**DOI:** 10.1186/s12992-018-0325-1

**Published:** 2018-01-18

**Authors:** Reshman Tabassum, Guenter Froeschl, Jonas P. Cruz, Paolo C. Colet, Sukhen Dey, Sheikh Mohammed Shariful Islam

**Affiliations:** 1 Department of Management, Faculty of Business and Law, Deakin University, Melbourne, Australia; 2 Center for International Health, Medical Center of the University of Munich (LMU), Munich, Germany; 3 Division of Infectious Diseases and Tropical Medicine, Medical Center of the University of Munich (LMU), Munich, Germany; 4 Shaqra University, Shaqraa, Saudi Arabia; 5 Bellamarine University, Kentucky, USA; 6 Non-Communicable Diseases Initiative, International Center for Diarrhoeal Diseases Research, Bangladesh (ICDDR,B), 68, Shaheed Tajuddin Ahmed Sarani, Mohakhali, Dhaka, 1212 Bangladesh; 7 The George Institute for Global Health, University of Sydney, Sydney, NSW Australia; 8 Institute for Physical Activity and Nutrition (IPAN), School of Exercise and Nutrition Sciences, Faculty of Health, Deakin University, Geelong, Australia

**Keywords:** Chronic diseases, Health literacy, Behaviour change, Communication, Mass media

## Abstract

In recent years, non-communicable diseases (NCDs) have become epidemic in Bangladesh. Behaviour changing interventions are key to prevention and management of NCDs. A great majority of people in Bangladesh have low health literacy, are less receptive to health information, and are unlikely to embrace positive health behaviours. Mass media campaigns can play a pivotal role in changing health behaviours of the population. This review pinpoints the role of mass media campaigns for NCDs and the challenges along it, whilst stressing on NCD preventive programmes (with the examples from different countries) to change health behaviours in Bangladesh. Future research should underpin the use of innovative technologies and mobile phones, which might be a prospective option for NCD prevention and management in Bangladesh.

## Background

Non-communicable diseases (NCDs) are increasing in epidemic proportion in many developing countries, such as Bangladesh [1, 2]. Evidence suggests a higher age-specific mortality for NCDs among Bangladeshis compared to Western populations, which puts an enormous burden on healthcare systems [1, 3]. Adopting healthy lifestyles and facilitating behavioural changing tools are key to prevention and management of NCDs [4, 5]. However, a high proportion of Bangladeshi adults have low health literacy, poverty, and lack of knowledge and skills [6, 7]. These people often engross in a veritable sea of health-related news from many diverse sources, often without the means to distinguish what is really useful based on grounded or professional evidence and to understand the true significance of the intended messages.

Low health literacy may have adversarial health effects by limiting patients’ ability to grasp health information, follow prescribed medical instructions, and communicate with health professionals and to acquire proper and timely care [8]. Individuals with lower health literacy are more likely to engage in negative health behaviours, such as smoking, drinking, unhealthy diet, taking extra salt [9], and substances abuse and are prone to have poor health, and impaired medication management capacity; leading to uncontrolled chronic conditions [10], increased healthcare costs [11], hospitalisation, and mortality [12].

Mass media campaigns, including newspapers and other printed material, radio, television, billboards, etc., have been recognised as the main source of health information at the individual level [13]. The impact of this coverage on individual’s knowledge, perceptions and attitudes are impressive and are useful in disseminating information and forming public opinion; nevertheless, the possibility for distortion from inaccurate information is noteworthy worldwide [14]. Harnessing the benefits of mass media, many countries have been successful in promoting healthy behavioural change programmes and improving health literacy, for example, Meena cartoons in Bangladesh. The Government of Bangladesh has recently stressed on preventing the epidemic of NCDs in Bangladesh [15]. However, the impact of mass media in changing health behaviours for prevention and management of NCDs is not well documented in Bangladesh. Hence, this review aims to discuss the role of mass media campaigns for NCDs and the challenges along it, whilst accentuating on NCD preventive programmes (with the examples from different countries) to change health behaviours in Bangladesh. The strategic mass media routes can endorse the development of future NCD prevention and management programmes in Bangladesh.

## Main text

Mass media campaigns have a broad reach and the potential to influence target audiences’ attitude towards healthy behavioural changes [13]. Over the preceding years, media campaigns have been striving to deliver health messages with the intention of changing health behaviours amongst defined populations [16]. In Bangladesh, mass media campaigns have been successful in increasing childhood immunisation, and reducing tobacco use and can potentially cover prevention of cardiovascular diseases, diabetes, cancer screening, and other NCDs. Such campaigns have customised health messages in media to reach target groups and to create maximum exposure to consumers, for instance, using broadcast media-television or radio, as well as outdoor media, including billboards, posters, and print media; thus disseminating significant health messages [16]. Disclosure of such health messages is mostly reflexive, resulting from an accompanying consequence of repetitive use of media. Regardless of the magnitude of mass media campaigns in changing health behaviours around the world, the use of mass media for prevention and management of NCDs remains significantly low in Bangladesh.

Health communication campaigns have been incorporating strategies to distribute messages designed—directly or indirectly— to enlighten, influence, and motivate target audiences’ attitudes on changing or maintaining health-related behaviours. Messages can be conveyed through a range of channels, including traditional mass media (e.g., television, newspapers); digital media, for example, social media (e.g., Facebook, Twitter, web forums); small print media (e.g., posters, brochures); community interactions (e.g., public forums); and personalised communications (e.g., hotline advising). Nevertheless, traditional mass media has prospects to sway individuals unswervingly and to transmit behaviour changing messages quicker than other communication approaches [16].

A variety of mass media campaigns unveils the prospective of being effective in diverse settings. For example, the SunSmart campaign for skin cancer prevention in Australia substantially increased sun protection behaviours among target groups or communities [17]. A previous study on 10 developing countries reported that television ads, which graphically portrayed  the health hazards of tobacco use, were likely to be effective among smokers [18]. A decade of anti-tobacco quit campaign with nationally coordinated Quitline service in Australia has led to significantly reduced smoking prevalence among adolescents and adults [19, 20]. The Florida “Truth” campaign used television and radio commercials, along with other media channels to inform the public about harmful effects of tobacco and to promote social penalties for smoking. Furthermore, The Safe Kids campaign is a global campaign that has used mass media to raise consciousness about the importance of wearing helmets and abiding road safety, fire safety, and home safety regulations to preclude injury and deaths. Such health communication campaigns are envisioned to sway decision-making procedures at both discrete and individual levels. Three anticipated outcomes of health campaigns are: lowering the impediments to change; facilitating individuals to embrace healthy lifestyles or to identify unhealthy social customs; and attaching valued sentiments with behavioural changes [16]. However, most of these programmes have been implemented in developed countries.

Many developing nations have also demonstrated successful mass media campaigns to improve health literacy and to change health behaviours. For instance, mass media campaigns for oral rehydration saline and breastfeeding resulted in reduced number of child mortality in Bangladesh [21], and anti-tobacco campaign in Bangladesh and Tongo led to decrease in smoking prevalence [22, 23]. Other examples include, oral cancer awareness campaigns in Malaysia [24], and a nationwide population screening campaign for diabetes in Brazil [25]. A systematic review evaluated 11 mass media campaigns in developing countries for diarrhoeal diseases, immunisation, and nutrition education and reported that mass media-centric campaigns positively influenced healthly behavioural changes among target audiences [21].These studies provide evidence that mass media can successfully change behavioural risk factors associated with NCDs in developing countries and should be integrated into NCD prevention and management programmes in Bangladesh.

Mass media channels are often more cost-effective at reaching people than any other communication methods. A previous systematic review demonstrated that mass media campaigns that were carefully planned and well executed, attained adequate audience exposure; intertwining with other ongoing prevention activities, such as high visibility enforcement [26]. A mass media education campaign for salt reduction was the most cost-effective population based intervention in both Vietnam and Argentina [27, 28]. Another study showed that the cost-effectiveness ratios for a mass media campaign in preventing the onset of smoking was economically more effective compared to other preventive and therapeutic strategies [29].

There is a need to emphasise on developing and monitoring the implementation of health campaigns to ensure that the interventions reach the target population. In recent years, information technology has played a great role to deliver health information using several technologies, including websites, social media, and mobile phone text messaging [30, 31]. A recent study in Bangladesh unveiled that mobile phone text messaging could reach a large number of patients with type 2 diabetes and could help to improve disease management at low cost [32, 33]. It is essential to underpin the use of innovative technologies and mobile phones, which might be a prospective option for NCD prevention and management in Bangladesh.

Several challenges impact the successful use of mass media campaigns in developing countries similar to Bangladesh (Table 1).

**Table 1:** Challenges for mass media in developing countries, including Bangladesh

1. Lack of urgency of people [7]; due to ignorance and misconception [34],
2. Inadequate information [1], poor health education, and lack of enthusiasm; due to low health literacy [35],
3. Absence of evidence based mass media programmes to support NCD prevention,
4. Issues with dissemination of right message using appropriate media [36],
5. Technological issues using digital media, i.e. privacy concerns, and cyber security risks [37, 38],
6. Alteration of messages [39].

## Conclusion

Mass media has enormous potential to support healthy behavioural changes and should be integrated into the NCD prevention programmes in Bangladesh. Bangladesh needs to pledge for effective measures through strategic routes, while planning to assimilate additional evidence on preventing and managing NCDs with appropriate mass media channels. Further research on emerging digital technologies necessitates to be explored in developing countries, such as Bangladesh, for changing health behaviour towards NCDs among target population.
